# Oral microbiome-SASP-aging axis: mechanisms and targeted intervention strategies for age-related diseases

**DOI:** 10.1080/20002297.2026.2616138

**Published:** 2026-01-15

**Authors:** Enfang Wu, Xueyu Li, Zichen Ni, Feng Zhao, Chen Jia

**Affiliations:** a Department of Disease Prevention and Control, General Hospital of Northern Theater Command, Shenyang, China; b General Hospital of Northern Theater Command, Shenyang, Liaoning, China; c The Forth Clinical College of China Medical University, Shenyang, China

**Keywords:** Oral microbiome, senescence-associated secretory phenotype, aging-related diseases, small molecule inhibitors, probiotics, traditional Chinese medicine

## Abstract

**Background:**

Global demographic aging is intensifying the burden of age-related diseases. Cellular senescence and the accompanying senescence-associated secretory phenotype (SASP) act as key drivers of disease progression by mediating chronic inflammation. As the second largest microbial community in the human body, the oral microbiome occupies a central position in systemic aging pathologies, and its dysbiosis and interaction with SASP are critical in this process. An imbalanced oral microbiota contributes to systemic chronic conditions via metabolic activities, virulence factor release, and immune system activation, while SASP serves as a central molecular mediator linking microbial dysbiosis to chronic inflammation, with well-recognized involvement in inflammatory bowel disease, bone disorders, and neurodegenerative conditions.

**Objective:**

This review aims to examine the mechanism by which oral pathogens directly modulate SASP secretion via microbial metabolites and virulence factors to drive the pathogenesis of age-related diseases, propose a unifying framework of the ‘oral microbiome-SASP-aging’ axis, summarize therapeutic interventions targeting this axis, and suggest future development directions for precise modulation of the ‘microbiome-SASP-aging’ cascade.

**Design:**

A narrative review was conducted to synthesize and analyze existing literature on the interplay between the oral microbiome, SASP, and age-related diseases. The review focused on mechanisms of oral pathogen-mediated SASP modulation, therapeutic strategies targeting the ‘oral microbiome-SASP-aging’ axis, and potential advancements in precise therapeutic delivery and combinatorial therapies.

**Results:**

The ‘oral microbiome-SASP-aging’ axis serves as a unifying framework for these pathologies. SASP inhibitors, probiotics, and traditional Chinese medicine (TCM) targeting this axis show promise for age-related disease management. Additionally, spatiotemporally precise delivery systems and probiotic-TCM combinatorial therapies are proposed for precise modulation of the ‘microbiome-SASP-aging’ cascade.

**Conclusions:**

The ‘oral microbiome-SASP-aging’ axis is a pivotal pathway driving age-related diseases. Therapeutic strategies targeting this axis hold significant promise for clinical management of these diseases. Future advancements in spatiotemporally precise delivery systems and combinatorial therapies are anticipated to enable precise modulation of the ‘microbiome-SASP-aging’ cascade, offering novel avenues for the prevention and treatment of age-related diseases.

## Introduction

**Figure 1.**
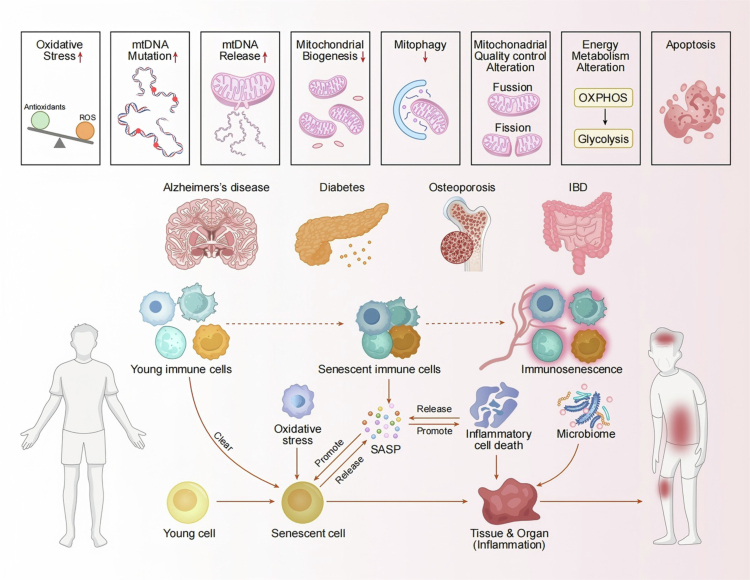
Oxidative stress precipitates mitochondrial dysfunction as well as metabolic reprogramming, which collectively promote DNA damage and mutation accumulation. These phenomena facilitate the transition of youthful cells into senescent cells, characterized by the secretion of factors associated with the SASP. The SASP exacerbates inflammatory responses, induces apoptosis in adjacent cells, and disrupts tissue/organ homeostasis. Concurrently, microbial dysbiosis – particularly an imbalance within the oral microbiota – intensifies oxidative stress and SASP secretion via inflammatory signaling pathways, thereby establishing a reciprocal feedback loop. Collectively, these pathological processes contribute to the onset of age-related diseases such as Alzheimer’s disease, diabetes mellitus, osteoporosis, and IBD. SASP inhibitors may intervene in this cascade by alleviating oxidative stress and consequently mitigating cellular senescence along with disease progression; SASP: senescence-associated secretory phenotype; IBD: inflammatory bowel disease.

In recent years, the escalating global burden of population aging has spurred efforts to develop strategies that extend the health span and address the health challenges of older adults. Aging is an inevitable biological process characterized by the progressive decline of tissue and organ function, metabolic dysregulation and altered metabolite concentrations, all of which elevate the risk of age-related diseases [1]. Cellular senescence is a fundamental hallmark of aging. Triggered by diverse stressors, this process is defined by irreversible cell cycle arrest and the development of a complex senescence-associated secretory phenotype (SASP) [2,3]. The accumulation of senescent cells exerts harmful effects on the tissue microenvironment, including promoting inflammation and tissue dysfunction, thereby playing a unique role in systemic metabolic dysfunction and various age-related pathologies [4]. The oral microbiome is hailed as the second largest microbial community in the human body and serves as the ‘second gut’ microbial reservoir for human aging. It features a highly diverse ecosystem comprising bacteria, fungi, and viruses [5]. To date, it has been discovered that the oral microbiome significantly influences host systemic and oral health by modulating metabolic and immune pathways [6]. Recent attention has focused on the crosstalk between cellular senescence and oral microbiome dysbiosis and its consequences for host health. While evidence indicates that the oral microbiome can accelerate disease progression by stimulating SASP-mediated systemic chronic inflammation, the intricate nature of their interactions and their collective impact on host aging remain unclear. Here, we first explored the correlation between the oral microbiome and aging. Then, we systematically summarized how the oral microbiome promotes the progression of aging-related diseases through the secretion of SASP components to induce chronic inflammation. Finally, we discussed the efficacy of therapeutic measures targeting the SASP in diseases (Figure 1). Unlike systematic reviews or meta-analyses, a formal PRISMA-compliant search protocol was not employed; instead, we focused on integrating key conceptual advances, mechanistic insights, and representative studies to construct a comprehensive theoretical framework.

## Cellular senescence and SASP

In 1961, Hayflick et al. [7]. first described the phenomenon of stable, irreversible growth arrest in normal human cells, terming it ‘cellular senescence’ and identifying it as a fundamental mechanism of aging. Biomarkers that arise during cellular senescence, such as DNA damage, telomere shortening, increased oxidative stress, and the expression of the key cell cycle factors p16^Ink4a^ and p21^Cip1^, have become critical therapeutic targets for age-related chronic diseases [8]. For example, using telomerase activators, drugs that selectively kill senescent cells [9], and methods that trigger p16^Ink4a^ expression induction systems [10] can eliminate senescent cells and improve aging signs in mice [11]. The SASP concept, first formalized by Campisi et al. in 2008, describes an intrinsic aging phenotype with a significant role in chronic inflammation and tissue degeneration [12]. SASP refers to the mediators secreted by senescent cells into the local and systemic tissue microenvironment under stress conditions, including various growth factors (TGFβ) [13], chemokines (CXC chemokine ligands 1/2 [CXCL1/CXCL2] [14]), pro-inflammatory cytokines (IL-1α, IL-1β, IL-11 [15] and TNF-*α* [16]), proteases (MMPs) [17] and extracellular vesicles [18]. Consequently, the functional influence of the SASP is dualistic, as it can promote cell growth and suppress tumors by recruiting immune cells to clear senescent cells [19], but it can also induce a state of systemic chronic low-grade inflammation (inflammatory aging) that accelerates aging [20]. This depends on the individual’s age; in young individuals, the SASP typically facilitates tissue repair and tumor suppression; however, as age increases, the chronic accumulation of SASP factors due to the accumulation of senescent cells becomes a key trigger for various aging-related pathological features [21]. Supporting this, heterochronic parabiosis studies have shown that blood from aged mice induces aging characteristics in young mice, indicating the transmissible nature of aging factors [22]. The elimination of SASP accumulated from senescent cells improves mouse health, highlighting the potential benefits of rejuvenation therapies [23]. Notably, diseases related to age and pathological changes associated with aging are not unique to the elderly. Unlike chronological aging, biological aging causes age-related diseases to start from early adulthood in some individuals. Chronological age provides a baseline for disease risk prediction, but biological age captures the ‘actual aging state’ that mediates pathological progression. An increase in SASP levels in individuals of the same actual age leads to the earlier onset of age-related diseases, which is related to the gradual accumulation and interaction of biological aging characteristics [17]. These inter-individual differences highlight the urgent need to utilize multi-omics technologies to identify specific biomarkers related to aging for personalized medical strategies. The epigenetic clock, which quantifies DNA methylation changes related to age and has become a reliable tool for assessing biological age, is more accurate than simple age calculation in reflecting an individual's functional aging status [24].

The understanding of aging is an expanding concept. Chronic inflammation and microbiota dysbiosis are recognized as two key factors in the human aging process [25]. Alterations in the microbiota’s composition and function are intimately linked to aging. There is growing evidence highlighting that bidirectional host‒microbiome communication is essential for normal physiological function. Therefore, dysbiosis can lead to various pathological conditions in the host, including chronic inflammation and disease progression [26].

## Oral microbiota and SASP

The production and secretion of SASP factors are precisely regulated by a complex network of internal and external factors, far beyond the sole influence of the oral microbiota. Internal regulatory factors include DNA damage induced by oxidative stress and other stimuli, which directly activates the DNA damage response pathway to prompt senescent cells to secrete pro-inflammatory factors such as IL-6 and TNF-*α* [27]; telomere attrition which triggers replicative senescence and sustains SASP secretion through downstream signaling cascades involving p53 and p16^Ink4a^ [28]. Oral microbiota imbalance is an important link in this regulatory network, but it works in synergy with intestinal microbiota imbalance, metabolic stress and environmental exposure, collectively forming an ‘SASP regulatory network’ that accelerates physiological aging and pathological diseases. In addition to the direct induction of senescence in epithelial or immune cells, oral microbiota dysbiosis modulates the secretion of SASP and aging by disrupting the function of tissue-resident mesenchymal stem cells, a key cell population for tissue homeostasis and regeneration [29]. The oral microbiota and its host environment are mutually dependent relationship [6]. Typically, a state of homeostasis prevails between the microbial community and the host, which supports normal tissue and immune system development. However, deterioration of the host environment (e.g. due to injury or inflammation) can disrupt the oral microbiome’s ecological balance, which is characterized by a reduction in beneficial microbes and an increase in pathogens, thereby promoting the progression of disease. In fact, oral microbiota dysbiosis is considered a direct precursor to common oral diseases such as dental caries and periodontitis [30]. Oral microorganisms can translocate into the gastrointestinal tract via saliva and food or enter the bloodstream through wounds, thus instigating systemic inflammation and age-related diseases. Accordingly, oral microbiota dysbiosis is implicated in the progression of numerous systemic conditions. Research has shown that periodontitis, one of the most common age-related inflammatory diseases [31], coexists with many inflammatory diseases, such as inflammatory bowel disease, diabetes, and AD [32]. The intricate interactions between senescent cells and immune cells mediated by the SASP are shown in Table 1.

**Table 1.** The intricate interactions between senescent cells and immune cells mediated by SASP.

Immunosuppressive	Immune cells type	Species of repressed SASP	References
	CD4^+^ T, CD8^+^ T	CXCL1, CXCL2, IL-6	[33,34]
	Treg	TGF-β	[35]
	NKT	CCL-2	[36]
	MDSC	CCL2, CCL6, CCL8, CCL11, CSF-1, IL-1IL-6, IL-10, CXCL1, CXCR2, MMP3	[37]
	M2 macrophage	IL-6, IL8,1L-10, IL-13, CCL17.CCL2 CXCL1, CXCL2, CXCL3, CXCLB, PGE2, MMP3, MCP-1, M-CSF, CSF-1	[38,39]
Immunosupportive	CD4^+^ T, CD8^+^ T	CXCL1, CXCL2, 1L-1α, IL-6, IL-29, IL-28a, IL-28b, IFN-γ	[40]
	NKT	CSF1, MCP1, CXCL1, CXCL10, IL-15, CCL2, CCL3, CCL4, CCL5	[41]
	M1 macrophage	IL-1β, IL-6, TNF-α, MCP-1, IFN-γ	[42,43]

Similar to the gut microbiome, the human oral microbiome undergoes dysbiosis with advancing age [44]. This progressive change is closely associated with both local and systemic inflammatory aging [45]. Age-related declines in physiological function, waning immunity, weakened oral mucosal barrier function, reduced saliva secretion, and diminished oral self-cleaning capacity collectively contribute to dysbiosis and the proliferation of pathogenic microbes within the oral microenvironment. The release of pro-inflammatory factors in the oral cavity can disrupt systemic homeostasis [46], and the host’s systemic state, in turn, influences the oral microbiota. For example, during cellular senescence, reduced IgA secretion in B cells in cervical lymph nodes promotes the expansion of oral pathogenic bacteria [47]. SASP regulation exhibits temporal heterogeneity, influencing different stages of aging and the aging process. Signaling pathways such as the NF-κB and C/EBPβ pathways promote the initial SASP, while LINE1 drives IFN regulation of late-stage aging in senescent cells [48]. NOTCH1 signaling helps determine whether a cell secretes TGF*-*β secretion or undergoes C/EBPβ-mediated pro-inflammatory secretion [49]. The effects of the SASP are also environment-dependent. In a long-term glucocorticoid-induced osteoporosis model, adipocyte aging SASP secondary feedback induces further cellular senescence [50], whereas activation of Notch signaling reduces SASP effects and inhibits secondary aging in neighboring cells [51]. This functional duality positions the SASP as a potential biomarker for cellular senescence [52]. The aging process forms an oral microbiota–SASP–aging axis. Oral pathogens directly exacerbate the SASP. In mouse models, *P. gingivalis* exacerbates the senescence of surrounding dendritic cells via exosomes [53]. *F. nucleatum* further promotes SASP secretion by esophageal squamous cell carcinoma cells after chemotherapy, which alters their drug resistance and cancer progression [54]. *Aggregatibacter actinomycetemcomitans* releases large amounts of inflammatory factors, such as IL-6, IL-8, RANKL, and toxins, exacerbating periodontitis [55]. Simultaneously, elevated SASP disrupts the oral-gut microbiota, thus creating a positive feedback loop that further drives aging propagation [56].

## The role of oral microbiota-mediated SASP in age-related diseases

**Figure 2.**
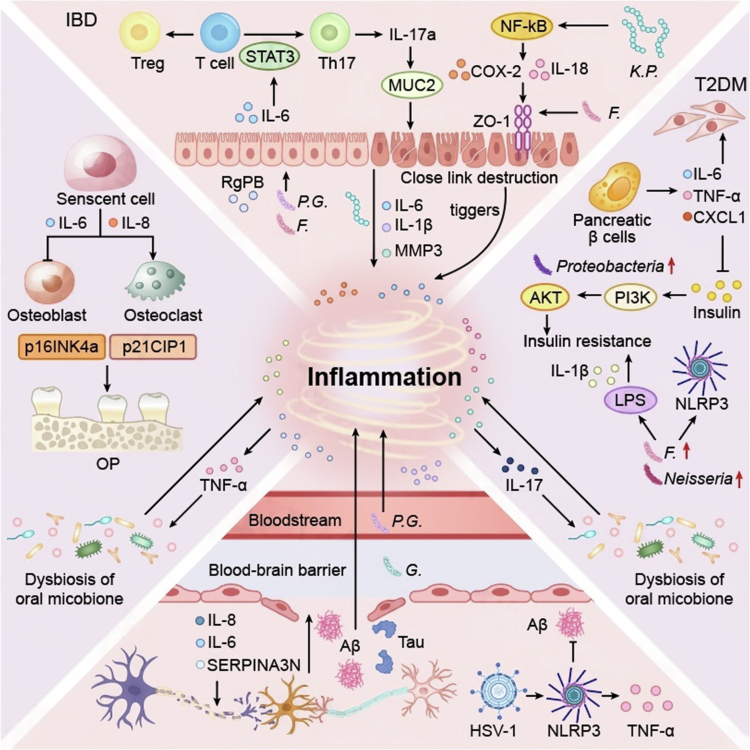
This figure delineates the tissue-specific pathogenic cascades through which oral microbiota dysbiosis regulates the secretion of SASP. Inflammatory Bowel Disease: Oral pathogens disrupt intestinal epithelial barrier integrity by degrading tight junction proteins and activating the NF-κB/STAT3 signaling pathways. SASP factors further skew the Th17/Treg immune balance and degrade the intestinal mucosal extracellular matrix, collectively exacerbating intestinal inflammation and barrier dysfunction. AD: Porphyromonas gingivalis traverses the blood‒brain barrier, releasing gingipains that directly cleave Tau protein; its metabolite phosphoglycerol dihydroceramide induces Aβ production and triggers SASP secretion from senescent microglia and astrocytes. Additionally, HSV-1 infection activates the NLRP3 inflammasome, further amplifying neuroinflammation and cognitive impairment. T2DM: Oral microbiota dysbiosis characterized by elevated LPS levels, which activate the NLRP3 inflammasome to promote IL-1β secretion. SASP derived from senescent pancreatic β-cells inhibits insulin signaling via disruption of the PI3K/AKT pathway, while gingipains degrade insulin receptors; these two processes synergistically induce systemic insulin resistance. Osteoporosis: SASP secreted by senescent osteocytes and osteoblasts disrupts the RANKL/OPG axis, promoting osteoclast differentiation and suppressing osteogenic activity. Oral pathogens exacerbating systemic bone loss through TNF-*α* mediated inflammation. HSV-1: herpes simplex virus type 1; T2DM: Type 2 diabetes mellitus; LPS: lipopolysaccharide; TLR2: Toll-like receptor 2; AD: Alzheimer’s disease; Aβ: amyloid-β.

Research on the relationship between oral microbiome dysbiosis and various age-related diseases is increasing. The inflammatory phenotype associated with aging may contribute to the development of chronic diseases. As noted previously, oral microbiota dysbiosis is closely associated with the secretion of SASP factors by senescent cells. However, the specific mechanisms underlying this relationship require further clarification. Therefore, comprehending and proactively preventing oral microbiome dysbiosis and the accumulation of SASP components are essential for promoting healthy longevity. In this section, current advances in understanding the crosstalk between the oral microbiome and SASP in age-related diseases are synthesized (Figure 2). Age-related diseases are not only related to the oral microbiota, but also influenced by various lifestyle factors, including smoking, physical activity, diet, and other environmental and lifestyle changes [57]. Coupled with population aging, these factors are generally considered to be the reasons for the rapid increase in the prevalence and incidence of type 2 diabetes worldwide in recent decades [58]. The quality and quantity of diet, insufficient physical activity, prolonged exposure to electronic screens or prolonged sitting, exposure to noise or dust, insufficient sleep or disrupted sleep, smoking, stress and depression, as well as low socioeconomic status, all these factors can lead to an increase in the body mass index, resulting in the loss of β-cell function. Malnutrition can cause a disordered microbial community structure, leading to an unfavorable microbiota state and subsequently triggering inflammatory responses [59]. Plant chemicals in exercise and diet activate the cell defense and anti-inflammatory pathways through the Nrf2 signaling pathway, which has a direct beneficial effect on β-cell function [60]. Together, they become protective factors for preventing diabetes. With respect to five low-risk lifestyles, studies have shown that the expected lifespan of women at the age of 50 may be extended by 14.0 years and that of men by 12.2 years. Those who do not adopt these factors show even more significant effects [57]. Notably, diseases related to age and pathological changes associated with aging are not unique to the elderly – they can begin as early as in youth. Long-term exposure to risk factors such as persistent oral microbial imbalance and unhealthy lifestyles can lead to premature cell aging and abnormal secretion of SASP, laying the pathological foundation for diseases such as T2DM and AD in later life.

### Inflammatory bowel disease

Inflammatory bowel disease (IBD) is chronic gastrointestinal inflammation closely associated with microbiome alterations or dysbiosis [61]. Studies have shown that IBD patients often exhibit reduced gut microbiota diversity alongside markedly elevated rates of abnormal colonization by the oral microbiota (e.g. *P. gingivalis*, *F. nucleatum* and *Klebsiella pneumoniae*) in the gut [62,63]. Animal experiments confirmed that *F. nucleatum*, after entering the gut via swallowing, can directly compromise the epithelial barrier by degrading tight junction proteins such as occludin and zonula occludens-1, thus inducing intestinal mucosal immune imbalance [64,65]. The SASP acts as a key molecular bridge linking oral microbiota dysbiosis to IBD [66,67]. Studies have shown that SASP factors (IL-6, IL-1β and MMP3) released by senescent intestinal epithelial cells and immune cells exacerbate intestinal inflammation. For instance, IL-6 promotes Th17 cell differentiation through STAT3 pathway activation, disrupting the Th17/Treg balance [68]. MMP3 degrades intestinal mucosal matrix proteins, increases intestinal permeability, and promotes ulcer formation, which thus serves as an independent risk factor for IBD recurrence [69]. Notably, *P. gingivalis* LPS, a key bacterial metabolite, upregulates SASP secretion through a partially TLR4-dependent pathway primarily mediated by resident immune cells such as dendritic cells, thereby establishing an ‘oral microbiome–SASP–inflammation’ positive feedback loop [70]. In contrast to gut-derived Gram-negative bacterial LPS (e.g. *E. coli* LPS), *P. gingivalis* LPS induces a distinct SASP cytokine profile characterized by Th2-polarizing factors and operates independently of canonical TLR4 signaling, underscoring its functional specificity [70,71].

In addition, the oral microbiome contributes to IBD pathogenesis through specific mechanisms. For instance, *P. gingivalis*-derived virulence factors or gingipains (such as Arg-gingipain B) exacerbate colitis by regulating linolenic acid metabolism to enhance IL-17a expression or by cleaving MUC2 to disrupt the intestinal mucus barrier [72,73]. Similarly, the outer membrane vesicles of *F. nucleatum* activates the FADD-RIPK1-caspase3 pathway to induce abnormal autophagy in intestinal epithelial cells. Furthermore, the imbalance of its metabolic byproduct butyrate imbalance reduces the energy supply to colonic cells, exacerbating mucosal damage [74–76]. Furthermore, *Klebsiella pneumoniae* activates NF-κB to upregulate the expression of COX-2 and IL-18, reducing tight junction protein expression and thus exacerbating colitis in murine IBD models [77]. Franzin et al. [78]. found that *Klebsiella pneumoniae* diminishes its cytotoxicity and active metabolite levels by intaking and bio-transforming thiopurine drugs (commonly used for IBD treatment), which may lead to treatment failure in IBD patients. These findings indicate that the oral microbiome drives IBD’s chronic inflammatory process not only by impairing the intestinal barrier and activating immune cell senescence and SASP secretion but also affect IBD treatment by reducing drug efficacy.

### Alzheimer’s disease

Alzheimer’s disease (AD) is a neurodegenerative disease whose pathological mechanisms are closely related to aging [79]. Recent studies have shown that senescent microglia and astrocytes activate key signaling pathways, such as NF-κB pathway, to drive the release of SASP components, which are characterized by factors such as IL-6, IL-8 and SERPINA3N. These SASPs components directly damage neurons, promote Aβ deposition, and induce Tau pathology but also disrupt the blood‒brain barrier (BBB), which form a vicious cycle of neuroinflammation that ultimately accelerates brain aging and AD pathological progression [80,81]. In addition, the pathological progression of AD is also associated with changes in the oral microbiome [82]. For example, the periodontal pathogen *P. gingivalis* enters the bloodstream through daily oral activities, crosses the BBB into the brain, and drives the progression of AD pathology through various mechanisms, including the release of virulence factors, direct nerve damage and Tau pathology, and the induction of Aβ production and inflammation [83,84]. Specifically, *P. gingivalis*-derived gingipains directly cleave the Tau protein to generate C-terminal toxic fragments and activation of caspase-3 to promote tau hyperphosphorylation and neurofibrillary tangle formation in AD [84]. Meanwhile, phosphoglycerol dihydroceramide, a novel *P. gingivalis*-derived virulence factor, promotes the production of Aβ and SASP factors (including *β*-galactosidase, cathepsin B, and proinflammatory cytokines [TNF-*α* and IL-6]), thereby exacerbating neuroinflammation [84]. Notably, gingipain inhibitors (COR271, COR286, and COR388) clear brain infections, inhibit Aβ production, and alleviate neuroinflammation, thus exerting neuroprotective effects in AD model mice [83,85].

Furthermore, the oral microbiome participates in AD pathological progression by mediating systemic inflammatory responses [86]. Serum antibodies against *F. nucleatum* are highly expressed in AD patient brains and promote microglial TNF-*α* release through nucleotide-binding oligomerization domain-like receptor family pyrin domain containing 3 (NLRP3) inflammasome activation, thus triggering inflammatory responses that worsen cognitive impairment [87,88]. Similarly, oral herpes simplex virus type 1 infection significantly affects the progression of AD. Wang et al. [89] reported that HSV-1 infection induces microglia recruitment to the viral core, triggering microglia phagocytosis of HSV-GFP-positive neuronal cells and activating the NLRP3 inflammatory pathway, thereby accelerating the progression of AD. Blocking NLRP3 inflammasome signaling effectively reduces Aβ deposition and alleviates cognitive impairments in diseased mice.

### Diabetes

Type 2 diabetes mellitus (T2DM) is one of the major contributors to the health burden of older adults. According to statistics, older adults (aged 65 years and older) account for nearly half of all diabetes patients [90]. Studies have shown that the oral microbiomes in elderly T2DM patients is predominantly *Firmicutes*, whereas *Proteobacteria* dominate in younger patients [91]. Even in T2DM patients, without oral diseases, the oral microbiome exhibits characteristic dysbiosis, characterized by an increased abundance of *P. gingivalis*, *F. nucleatum*, *Prevotella melaninogenica*, *Streptococcus* and *Weissella*, in addition to reducing *Capnocytophaga* and *Cardiobacterium*. This dysbiosis is significantly associated with glycemic control levels [92–94]. Research has indicated that oral microbiome dysbiosis and the SASP play a key regulatory role in the pathological progression of T2DM. Moreover, the SASP is the core molecular bridge linking microbiome dysbiosis and metabolic abnormalities. Aged β*-*cell-secreted SASP (e.g. IL-6, TNF-*α* and CXCL1) can directly inhibit insulin signaling or exacerbate systemic insulin resistance through endocrine pathways [95]. Recent studies have revealed that oral pathogens influence the pathological progression of diabetes by regulating the SASP. For example, *P. gingivalis* secretes gingipains that specifically cleave insulin receptors, blocking the insulin-dependent PI3K/Akt pathway and thereby inducing insulin resistance [96]. Similarly, Wang et al. [97]. found significantly increased *Neisseria* and *F. nucleatum* abundance in T2DM patient saliva. Mechanistically, these pathogens may activate the NLRP3 inflammasome and induce IL-1β secretion by increasing the concentration of LPS in the host microenvironment, thereby promoting insulin resistance in T2DM patients. Notably, a bidirectional relationship is observed between oral microbiome dysbiosis and T2DM. The hyperglycemic environment in T2DM patients elevates IL-17 levels, which in turn enhances oral microbiome pathogenicity [98]. Although the mechanism link between oral pathogens and NLRP3 inflammasome activation is mainly supported by *in vitro* and animal studies, the newly emerging human clinical data on the analysis of the microbial composition in the saliva of patients with type 2 diabetes and healthy individuals show that specific oral pathogens are associated with the prognosis of patients with type 2 diabetes. The relative abundance of the Fusobacteria and Campylobacteria phyla in the saliva of patients with type 2 diabetes is lower than that of healthy individuals, while the relative abundance of the Proteobacteria phylum is higher than that of healthy individuals [97,99].

### Osteoporosis

Osteoporosis (OP) is a common age-related disease characterized by decreased bone density and systemic bone microstructural disorders [100]. Studies indicate that disrupted oral microbial homeostasis is a significant pathogenic factor in osteoporosis. Compared with healthy individuals, periodontitis patients (exhibiting oral microbiome imbalance) face an increased OP risk. While OP patients show greater susceptibility to periodontitis [101–103]. Aging osteocytes secrete SASP factors (IL-6 and IL-8), which promotes osteoclast differentiation and inhibits osteoblast activity, leading to bone loss. The high expression of p16^Ink4a^ and p21^Cip1^ in the bone tissue of elderly women confirms the direct association between aging and osteoporosis [104,105]. The oral microbiome regulates bone metabolism and exhibits strong connections to osteoporosis [106]. Studies indicate that dysbiosis of the oral microbiome in periodontitis exacerbates bone loss in ovariectomized rats through TNF-*α* mediated systemic inflammation and tryptophan metabolism [107,108]. *F. alocis* is recognized as one of the key contributing pathogens rather than a classic keystone pathogen. Classic keystone pathogens (e.g. *P. gingivalis*) drive microbial dysbiosis and disease progression through manipulating the microbiome structure and host immune responses, even at low abundances [109]. In contrast, *F. alocis* exerts its pathogenic effects primarily through synergistic interactions with other periodontal pathogens and direct induction of host tissue damage without exerting a dominant regulatory role in microbiome homeostasis [110]. Nevertheless, its contribution to osteoporosis is non-negligible: EVs derived from *F. alocis* induces the production of proinflammatory cytokines (IL-6 and TNF-*α*), promotes osteoclastogenesis, and triggers systemic bone loss through TLR2 signaling [111]. which directly amplifies the SASP-related bone catabolic cascade. Collectively, *P. gingivalis* acts as a keystone pathogen driving the initiation and progression of periodontitis-associated osteoporosis, while *F. alocis* functions as an important contributing pathogen that synergizes with *P. gingivalis* and directly induces bone loss through TLR2-mediated pathways. Their combined effects amplify the 'oral microbiome-SASP-bone loss' cascade, highlighting the need for targeted intervention against multiple pathogenic taxa in clinical practice. However, understanding its mechanism of action, long-term safety and bioavailability remains an area for further research. In this context, physical exercise has emerged as an effective non-pharmacological approach, capable of directly regulating cellular aging and promoting tissue regeneration, preventing and treating age-related musculoskeletal diseases, reducing oxidative stress, improving mitochondrial function and regulating signaling pathways involved in energy metabolism and inflammatory responses [112]. The latest evidence shows that the exercise mimetic SLU-PP-332 can increase the expression of ERRα, confirming that the PGC-1α/ERRα axis, which is involved in regulating mitochondrial biogenesis and fatty acid oxidation [113]. Exercise promotes an increase in catalase and superoxide dismutase 2, confirming its effectiveness in promoting mitochondrial adaptation and enhancing muscle antioxidant capacity, which helps to counteract the effects of aging [114].

## Targeting the oral microbiota and SASP for the treatment of age-related diseases

### SASP inhibitors

**Figure 3.**
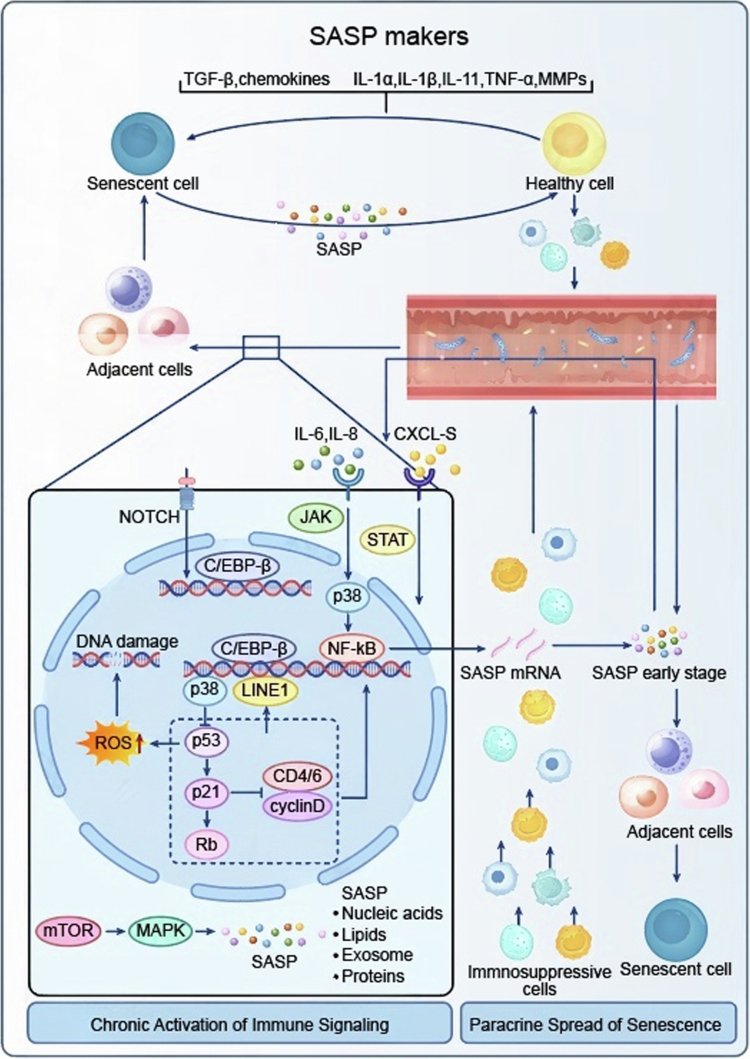
This figure illustrates the mechanisms of the senescence-associated secretory phenotype (SASP) production and its paracrine spread of senescence. Senescent cells secrete SASP markers, including cytokines such as TGF-β, IL-1α, chemokines and MMPs, which act on healthy cells, adjacent cells, and lead to the recruitment of immunosuppressive cells, driving the paracrine spread of senescence. Intracellularly, triggers such as DNA damage and ROS activate pathways such as the NOTCH, JAK/STAT, p38, NF-κB and mTOR/MAPK, along with key molecules such as C/EBP-β, p53 and LINE1, to regulate the production of SASP components, including nucleic acids, lipids, exosomes and proteins, ultimately resulting in chronic activation of immune signaling.

The SASP works as the core functional mediator for information exchange between senescent cells and their microenvironment. Its abnormal activation reshapes the local tissue microenvironment, exerting a decisive regulatory function in the onset and progression of various age-related diseases, including neurodegenerative diseases, osteoarthritis, and cardiovascular diseases [115]. Given the pathological driving effects of the SASP, small-molecule inhibitors and biopharmaceuticals that modulate the SASP represent a central research focus in aging biology and disease intervention. Systematic analysis of their mechanisms of action offers essential theoretical support for developing precision disease intervention strategies [116]. The dynamic regulation of the SASP relies on a complex signaling network, and targeted intervention of key molecular nodes has been proven to be an effective regulatory strategy. Inhibitors specific to different signaling pathways show therapeutic potential across various aging-related disease models (Figure 3).

NF-κB signaling, as a core transcription factor in the SASP regulatory network, sustains SASP persistence by mediating the transcriptional activation of pro-inflammatory factors such as IL-6 and TNF-*α* [117]. Specific inhibition of NF-κB activity significantly downregulates the secretion profile of pro-inflammatory factors, thereby blocking the cascade amplification of aging-related inflammation [118]. For example, dendrobine alleviates osteoarthritis pathological progression by inhibiting the expression of SASP factors such as IL-6 and MMP-13 in chondrocytes and aging phenotypes via the ROS/NF-κB axis [119]. Oridonin inhibits the expression of IL-1β and SASP and suppresses the NF-κB pathway via the PI3K/AKT pathway, which improves cartilage wear and alleviating OA progression in an ACLT rat model [120].

The JAK-STAT signaling pathway contributes to sustained SASP activation by mediating cytokine cascade signaling. Studies have shown that the JAK inhibitor ruxolitinib or the antiviral drug remdesivir not only breaks the ‘senescence-cytokine secretion’ vicious cycle but also restores the proliferation capacity of human umbilical vein endothelial cells by normalizing the expression of SARS-CoV-2 entry receptors, thereby demonstrating significant improvements in age-related inflammation [121]. Additionally, the JAK inhibitor peficitinib effectively improves 5-fluorouracil-induced intestinal damage by inhibiting the accumulation of senescent cells; downregulating the secretion of the inflammatory factors TNF-*α*, IL-1β and IL-6; and reducing oxidative stress, thus suggesting its potential for managing chemotherapy-related complications [122].

mTOR is a key molecular switch linking the cellular metabolic state to the aging process. The inhibitor rapamycin selectively inhibits SASP components by regulating the interaction between the MK2 kinase and 4EBP1. This mechanism explains its multifunctionality in cancer and age-related diseases and reveals the functional association between metabolic regulation and SASP heterogeneity [123]. Additionally, p38 mitogen-activated protein kinase (MAPK) directly participates in the initiation of the SASP by coupling cellular stress signals with transcriptional regulatory networks [124]. Forced expression of the MAPK pathway gene BRAF^V600E^ mutation in early-stage mouse and human hematopoietic progenitor cells (HPCs) induces senescence programs and SASP secretion. Rapamycin-mediated inhibition of the mTOR signaling pathway significantly reduces the release of SASP-associated molecules and attenuates the abnormal differentiation of BRAF-V600E^+^ HPCs, thus alleviating the pathophysiology of aging-driven Langerhans cell histiocytosis [125].

SASP regulation also modulates organelle interactions and stress signaling. Mitochondrial dysfunction, one of the core characteristics of senescent cells, participates in the activation of the SASP through nuclear-to-cytoplasmic retrograde signaling regulation [126]. Studies have shown that downregulation of nuclear-encoded mitochondrial oxidative phosphorylation genes triggers sustained activation of the ROS‒JNK signaling axis, thereby driving the release of cytoplasmic chromatin fragments and ultimately exacerbating the pathological effects of the SASP. While the HDAC inhibitor Trichostatin A effectively inhibits SASP secretion in senescent cells by reshaping mitochondrial–nuclear signaling communication, providing a new direction for SASP intervention targeting organelle interactions [127].

These studies collectively indicate that the multi-targeted nature of SASP inhibitors provides distinct advantages for cross-indication interventions in aging-related diseases. Furthermore, combined intervention strategies based on cross-regulatory signaling pathways may enhance therapeutic efficacy.

### Probiotics

Oral probiotics can effectively improve periodontal health by regulating the balance of the oral microbiota, enhancing the function of the oral barrier, and particularly by regulating the host's local and systemic immune responses (through inhibiting excessive inflammation). This action helps reduce systemic inflammation levels through the ‘oral-systemic’ axis, which indirectly inhibits inflammatory aging processes and the production of SASP in distant tissues. Currently, specific oral probiotic formulations represent a promising adjunctive approach for maintaining oral health and controlling oral inflammation (e.g. periodontitis), and their potential systemic anti-inflammatory and anti-aging benefits require further investigation.

In recent years, oral probiotics, especially genetically engineered strains, have provided new therapeutic approaches for intervening in cell aging-related diseases by targeting the regulation of the ‘microbiome‒host aging axis’ [128]. Genetically engineered strains achieve reprogramming of aging immune cells through precise delivery of immunomodulatory molecules. Engineered *Escherichia coli* Nissle 1917 (ECN) delivers IL-2 not only directly promoting the generation of Tregs to alleviate chronic intestinal inflammation in IBD, but also enhancing the abundance of SCFA-producing bacteria, such as *Lachnospiraceae*_NK4A136 and *Odoribacter*, thereby enhancing intestinal mucosal barrier integrity in an SCFA-dependent manner and blocking systemic SASP dissemination [129,130]. *Bifidobacterium* FTJS7K1/FTJS5K1, on the other hand, counters gestational diabetes by expanding regulatory immune cells (Treg, Tfr and Breg), rebalancing inflammatory cytokines (reducing TNF-α/IL-6 while elevating TGF-β/IL-10) [131]. Additionally, oral administration of *Lactobacillus plantarum* LP45 reduces the RANKL/OPG ratio, inhibits osteoclast activation, and decreases the accumulation of senescent osteoblasts, thereby conferring benefits in preventing osteoporosis [132]. These results suggest that oral probiotics fundamentally reshape immune cell aging phenotypes rather than merely suppressing inflammation, thereby breaking the cycle of immune aging.

### Traditional Chinese medicine

**Figure 4.**
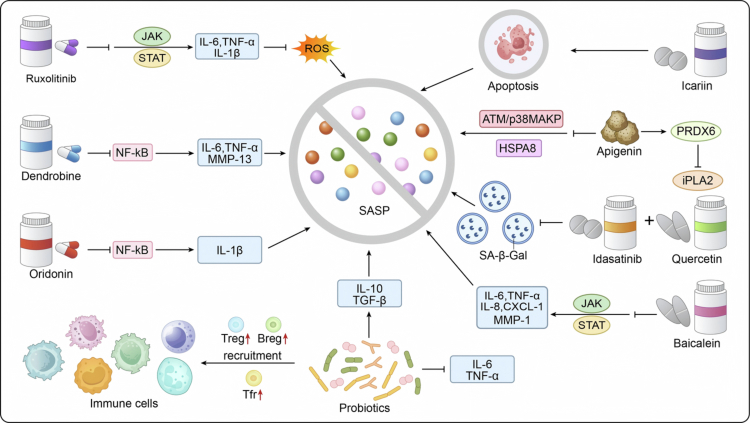
It depicts therapeutic strategies targeting the SASP. Various drugs like Ruxolitinib, Dendrobine, Oridonin, Icariin, Apigenin, Idasatinib, Quercetin and Baicalein act on signaling molecules such as JAK/STAT, NF-κB, ATM/p38MAPK, and targets like SA-β-Gal and PRDX6 to inhibit SASP production or induce the apoptosis of senescent cells. Additionally, immune cells such as Treg, Breg, Tfr and probiotics regulate the SASP through immune recruitment or cytokine modulation, with cytokines such as IL-6, TNF-*α* and IL-1β being key SASP components targeted in these processes.

Recent studies have revealed that various traditional Chinese medicines (TCMs) and their active components can effectively regulate the expression and secretion of SASP components, thereby intervening in the progression of related diseases and demonstrating unique therapeutic potential. These findings provide important TCM-based evidence and strategies for targeting the SASP in disease treatment (Figure 4).

Apigenin, a dietary source of flavonoids, has been identified as an anti-aging agent (senolytic) that blocks the interaction between ATM/p38MAPK and HSPA8, thereby preventing the transition of acute stress-associated phenotypes to chronic, harmful SASPs. Mechanistically, apigenin directly binds peroxiredoxin 6 (PRDX6), inhibiting its iPLA2 enzyme activity and derailing the pro-inflammatory cascade driving SASP establishment [133]. This precise targeting informs the rational design of next-generation SASP inhibitors.

In the field of neurodegenerative diseases, TCM components exhibit remarkable SASP-suppressing potency. For example, baicalein clearly protects against hydrogen peroxide-induced astrocyte senescence. Its mechanism involves inhibiting the secretion of key SASP factors (such as IL-6, IL-8, TNF-*α*, CXCL1 and MMP-1) and blocking the activation of the JAK2/STAT1 and NF-κB signaling pathways [134]. Additionally, baicalin has also an intervening effect on low-glucose-induced astrocyte senescence. The ethanol extracts of baicalensis significantly reduces the SA-*β*-gal positivity rate, decreases the secretion and mRNA expression of multiple SASP factors (such as IL-6, CXCL1, MMP-1, CXCL10, CXCL2 and CCL2), and alleviates cell cycle arrest. This study validated that this extract delays astrocyte senescence by targeting the CXCL10-mediated signaling axis, providing new insights into its mechanism of alleviating neurodegenerative diseases such as AD [135].

For metabolic disease-related cognitive impairment, the compound TCM Zishen Wanfang (ZSWF) operates through well-defined mechanisms. ZSWF administration suppresses Cdkn1a and SASP gene expression in cerebrovascular cells, enhances endothelial-pericyte communication, and inhibits high glucose-induced p21 and VCAM-1 expression in brain endothelial cells. Consequently, ZSWF preserves blood‒brain barrier integrity by countering vascular cell senescence and SASP release, thereby ameliorating diabetes-induced cognitive impairment [136].

In skeletal diseases, strategies targeting the SASP have also demonstrated therapeutic value. In a chronic high-altitude hypoxia (CHH)-induced osteoporosis mice model, the combined use of the known anti-aging drugs dasatinib and quercetin (DQ) has been proven effective. DQ treatment selectively eliminates senescent cells and significantly reduces SASP levels (as evidenced by decreased senescence markers in serum and bone marrow and weakened SA-*β*-Gal staining), effectively preventing the decline in bone density and bone mineral content in CHH mice, thereby demonstrating therapeutic benefits for osteoporosis [137]. Similarly, icariin activates the autophagy pathway, significantly reducing SASP expression in senescent macrophages and senescent bone marrow mesenchymal stem cells (S-BMSCs), alleviating inflammation, restoring the osteogenic function of S-BMSCs, and ultimately reducing bone loss in osteoporotic mice [138]. The synergistic effect of probiotics and herbal bioactive components is receiving increasing attention. Through multi-target and multi-pathway mechanisms, it forms a natural and systematic treatment strategy. It is particularly noteworthy that the fermentation of herbs by probiotics can produce a significant synergistic enhancement effect. During the fermentation process, the extracellular enzymes secreted by probiotics can hydrolyze the plant cell wall, promoting the release and transformation of active components [139]. For example, after fermentation by lactic acid bacteria, the content of curcumin in turmeric and its specific pharmacological activity have both been enhanced [140]. Similarly, when Angelica sinensis soup is co-fermented with *Lactobacillus plantarum*, its *α*-glucosidase inhibitory ability, DPPH free radical scavenging activity, and anti-glycation effect are significantly enhanced compared to the non-fermented samples [141]. On the other hand, the herbal matrix itself is rich in proteins, vitamins, and trace elements. These components provide a favorable nutritional environment for the growth, reproduction and metabolic activities of probiotics, further strengthening the activity and metabolic function of the microbiota. This bidirectional promotion relationship – probiotics optimize the release and transformation of herbal components, and herbal components support the survival and function of probiotics – constitutes a positive cycle, deepening the overall regulatory potential of the ‘microbiota‒host–metabolism’ axis and laying a theoretical basis for the development of the next generation of microecological preparations based on probiotic-herb co-fermentation [139].

### Nanotherapy

In recent years, nanotechnology has rapidly advanced in the medical field, leading to revolutionary breakthroughs to intervention strategies for aging-related diseases. Nanomaterials, with their unique physicochemical properties, including precise size control, functionalizable surface characteristics, excellent biocompatibility, and significant advantages in targeted delivery and enhanced efficacy, have become crucial force driving the development of precision medicine. The oral nanodelivery platform (QM@EP) has achieved integrated detection and treatment of inflammatory bowel disease [142] Novel antioxidant nanomaterials have demonstrated significant efficacy in colitis models through targeted delivery and the ability to scavenge reactive oxygen species [143]. The rise of nanotechnology has brought new hope for the development of IBD treatment drugs. Compared with conventional drug delivery systems, nanocarriers, owing to their unique properties, can effectively enhance the clinical efficacy of drugs and alleviate side effects [144]. Self-assembled nanoparticles based on short-chain fatty acids have shown the highest glucose tolerance in diabetes, with greater potential for therapeutic intervention [145]. In the field of bone metabolic diseases, the multifunctional nanosystem MSN-OI@Ce-TA effectively restores bone immune homeostasis by regulating macrophage epigenetic reprogramming and mitochondrial function [146]. Meanwhile, bone-targeted supramolecular nanostimulators based on natural products have opened up new directions for osteoporosis reversal [147]. For the complex pathological network of Alzheimer's disease, engineered nanovesicles (Gas6-NV-NPs) precisely regulate the neuroimmune microenvironment by restoring lysosomal function in microglia, enhancing phagocytic clearance, and promoting anti-inflammatory phenotypic transformation [148]. A peptide‒drug conjugate-based nanoplatform has been developed for immune–metabolic activation and in situ neural regeneration in advanced Alzheimer's disease [149]. Overall, nanomedicine is profoundly transforms the treatment paradigm of aging-related diseases through three dimensions: precise delivery systems, intelligent response mechanisms, and multi-target synergistic strategies. These advancements not only overcome the biological barrier limitations of traditional therapies but also lay a solid foundation for individualized precision medicine through spatiotemporal-specific microenvironmental regulation. Future research should further explore the long-term biological safety of nanomaterials, optimize their targeting efficiency, and clarify clinical translation pathways to promote the advancement of this frontier field into clinical practice.

## Conclusions and prospects

Cellular senescence and the subsequent SASP are the core pathogenic drivers of age-related metabolic disorders and their complications, making senescence-targeted therapeutic approaches a highly promising frontier in the management of aging-related diseases. This review systematically elaborates on the regulatory mechanisms of the ‘oral microbiota–SASP–aging’ axis in metabolic imbalance and therapeutic strategies targeting this axis, including SASP inhibitors, genetically engineered probiotics, active components of traditional Chinese medicine, and nanotherapies, which have shown significant efficacy in preclinical studies in modulating aging signaling cascades and restoring metabolic homeostasis. However, translating these preclinical advancements into clinically feasible interventions remains fraught with numerous unresolved challenges.

Firstly, there are significant gaps in both basic and translational research paradigms: the inherent heterogeneity of senescent cells in different tissues and disease contexts complicates the development of universal therapeutic strategies; there is a lack of standardized clinical endpoints and non-invasive biomarkers such as circulating SASP signatures and tissue-specific aging markers for quantifying senescent cell burden; pharmacokinetic/pharmacodynamic optimization of senolytic and senostatic agents, including dosing regimens, administration timing, and drug interactions, remains insufficiently addressed. Additionally, safety concerns pose a major obstacle: long-term use of senescence-targeted drugs may disrupt physiological processes and overlook potential carcinogenic risks. The lack of non-invasive *in vivo* imaging tools for senescent cells and the need for spatiotemporally precise drug delivery systems further impedes clinical translation.

To address these challenges, future research should prioritize several interrelated key directions. Firstly, advancing personalized and precision medicine frameworks: leveraging multi-omics technologies including genomics, microbiomics, proteomics and metabolomics, to interpret individual differences in aging trajectories, oral microbiota compositions and SASP signatures, which will facilitate the development of tailored interventions for specific patient subgroups, such as personalized probiotic formulations, targeted SASP inhibitor regimens, or precise periodontal treatments. Secondly, optimizing combination therapy strategies: rigorously exploring the synergistic combinations of senolytics, senostatics, antioxidants and immunomodulators, while systematically clarifying dose titration, administration timing, and sequence to maximize therapeutic efficacy and minimize adverse interactions. Thirdly, developing innovative technology platforms: integrating machine learning algorithms with novel tracing technologies (such as Sn – pTracer, Sn – cTracer) for non-invasive monitoring of senescent cell dynamics *in vivo*. Fourthly, standardizing aging biomarkers and clinical protocols: validating non-invasive, reproducible, and clinically accessible biomarkers such as circulating SASP factor profiles; microbial signature spectra; or epigenetic aging clocks should be validated to guide patient stratification, monitor treatment responses, and establish standardized clinical endpoints. Fifthly, promoting interdisciplinary collaboration: integrating insights from aging biology, microbiology, immunology, oncology, and clinical pharmacology to decipher the molecular crosstalk between oral microbiota dysbiosis, resident stem cell dysfunction, as emphasized in recent studies [29], and SASP secretion, thereby identifying new therapeutic targets and signaling nodes. Finally, ensuring rigorous safety assessment: conducting long-term clinical trials to evaluate the tolerability, durability, and long-term safety of senescence-targeted therapies, particularly in terms of immune function, metabolic homeostasis and carcinogenic risk, while promoting evidence-based public communication to manage expectations and reduce misinformation. In conclusion, targeting the ‘oral microbiota–SASP–aging’ axis holds great potential in transforming the clinical management of age-related metabolic disorders (Table 2).

**Table 2.** Targeting oral microbiota and SASP for the treatment of age-related diseases.

Types	Names	Relevant mechanisms	Models	Diseases	References
	Ruxolitinib or remdesivir	Inhibits the JAK-STAT signaling pathway; Normalizes the expression of SARS-CoV-2 entry receptors; Suppress the SASP, thereby restoring the proliferative capacity of human umbilical vein endothelial cells	Human umbilical vein endothelial cells	Improves age-related inflammatory diseases	[121]
	Peficitinib	Inhibits JAK signaling pathways and SASP factors TNF-*α*, IL-1β, and IL-6 secretion	Cell lines and BALB/C mice	Improves 5-fluorouracil-induced intestinal damage	[122]
	Rapamycin	Inhibits mTOR signaling pathway; Regulates the interaction between MK2 kinase and 4EBP1; Selectively inhibits SASP factors	Cell lines and mice	Inhibits the progression of age-related diseases	[123]
		Inhibits mTOR signaling pathway; Reduces the release of SASP-associated molecules and abnormal differentiation in BRAF-V600E + HPCs.	mouse	Attenuates the pathophysiology of Langerhans cell histiocytosis	[125]
	Trichostatin A	Inhibits SASP secretion in senescent cells by reshaping mitochondrial-nuclear signaling communication	Cell lines and mice	Inhibits the progression of age-related diseases	[127]
Probiotics	*Escherichia coli* Nissle 1917	Releases IL-2 to increase SCFA production, thereby enhancing intestinal mucosal barrier integrity; Blocks the systemic spread of SASP	C57BL/6 mice	Alleviate IBD	[129,130]
	*Bifidobacterium* FTJS7K1/FTJS5K1	Expands Treg, Tfr, and Breg cells, reduces TNF-*α* and IL-6, and increases TGF-*β* and IL-10	C57BL/6J mice	Prevents gestational diabetes	[131]
	*Lactobacillus plantarum* LP45	Reduces the RANKL/OPG ratio, inhibits osteoclast activation, and decreases the accumulation of senescent osteoblasts	Sprague–Dawley rats	Prevents osteoporosis	[132]
Traditional Chinese medicine	Apigenin	Blocks the interaction between ATM/p38MAPK and HSPA8; Targets PRDX6, inhibiting its pro-inflammatory iPLA2 enzyme activity, thereby disrupting the downstream signaling cascade driving SASP development	Cell lines and mice	Ameliorates age‐related conditions	[150]
	Baicalein	Inhibits the secretion of SASP factors (such as IL-6, IL-8, TNF-*α*,CXCL1, MMP-1) and blocks the activation of the JAK2/STAT1 and NF-κB signaling pathways	Human astrocytic cell line T98G	Alleviates astrocyte senescence	[134]
	Ethanol extracts of baicalensis	Reduces the SA-*β*-gal positivity rate, decreases the secretion of multiple SASP factors (such as IL-6, CXCL1, MMP-1, CXCL10, CXCL2, CCL2)	Human astrocytic cell line T98G	Alleviates neurodegenerative diseases such as AD	[135]
	Zishen Wanfang	Inhibits brain vascular cell senescence and SASP release	Diabetic mouse model	Improves diabetes-induced cognitive impairment	[136]
	Dasatinib and Quercetin	Selectively eliminates senescent cells and reduces SASP levels (weakened SA-*β*-Gal staining)	CHH-induced osteoporosis mice model	Improves symptoms of osteoporosis	[137]
	Icariin	Reduces SASP expression in senescent macrophages and S-BMSCs; Alleviates inflammation and restores the osteogenic function of S-BMSCs	Osteoporosis mouse model	Mitigates bone loss; Alleviates osteoporosis progression	[138]

RCT: Randomized controlled trial; TLR4: Toll-like receptor 4; SCFA: Short-chain fatty acid; IL-10: Interleukin 10; IL-8: Interleukin 8; TNF-α: Tumor necrosis factor-α; OVX: Ovariectomized Rats; Trp: Tryptophan; ZO-1: Zonula Occludens-1; SHP2: Src Homology 2 domain-containing protein tyrosine phosphatase 2; NF-κB: Nuclear factor kappa-B; IL-1β: Interleukin-1β. PRDX6: Peroxiredoxin 6; iPLA2: Independent phospholipase A2; AD: Alzheimer's disease; CHH: Chronic high-altitude hypoxia; S-BMSCs: Senescent bone marrow mesenchymal stem cell. SASP: Senescence-associated secretory phenotype; CXCL1: C-X-C motif chemokine ligand 1; MMP-1: Matrix metalloproteinase 1 IL-1β: Interleukin-1β; CXCL1: C-X-C motif chemokine ligand 1; MMP-1: Matrix metalloproteinase 1; CCL2: C-C motif chemokine ligand 2.
